# Hospital funding reforms in Canada: a narrative review of Ontario and Quebec strategies

**DOI:** 10.1186/s12961-022-00879-2

**Published:** 2022-06-27

**Authors:** Maude Laberge, Francesca Katherine Brundisini, Myriam Champagne, Imtiaz Daniel

**Affiliations:** 1 Department of Operations and Decision Systems, Faculty of Administration, Université Laval, 2325, rue de la Terrasse, Bureau #2519, Quebec City, QC G1V 0A6 Canada; 2 Vitam, centre de recherche en santé durable, Université Laval, Quebec City, Canada; 3 Centre de Recherche du CHU de Québec, Université Laval, Quebec City, Canada; 4 Institute of Health Policy, Management and Evaluation, University of Toronto Health Sciences Building, 155 College Street, Suite 425, Toronto, ON M5T 3M6 Canada; 5 Ontario Hospital Association, Toronto, Canada

**Keywords:** Implementation science, Patient-based funding, Hospital funding, Activity-based funding, Narrative review

## Abstract

**Background:**

In the early 2000s, Ontario and Quebec, two provinces of Canada, began to introduce hospital payment reforms to improve quality and access to care. This paper (1) critically reviews patient-based funding (PBF) implementation approaches used by Quebec and Ontario over 15 years, and (2) identifies factors that support or limit PBF implementation to inform future decisions regarding the use of PBF models in both provinces.

**Methods:**

We adopted a narrative review approach to document and critically analyse Quebec and Ontario experiences with the implementation of patient-based funding. We searched for documents in the scientific and grey literature and contacted key stakeholders to identify relevant policy documents.

**Results:**

Both provinces targeted similar hospital services—aligned with nationwide policy goals—fulfilling in part patient-based funding programmes’ objectives. We identified four factors that played a role in ensuring the successful—or not—implementation of these strategies: (1) adoption supports, (2) alignment with programme objectives, (3) funding incentives and (4) stakeholder engagement.

**Conclusions:**

This review provides lessons in the complexity of implementing hospital payment reforms. Implementation is enabled by adoption supports and funding incentives that align with policy objectives and by engaging stakeholders in the design of incentives.

## Background

Delivering accessible, cost-effective and high-quality healthcare is a critical endeavour for governments and health systems across the world. However, health system resources remain limited, and health decision-makers continue to explore alternative funding models to increase health services efficiency and quality of care. Canada is no exception, and has experimented with new funding models.

In Canada, healthcare is decentralized and under the responsibility of each province and territory (P/T). As such, P/T governments may design the organizational structure, resource allocation and the payment mechanisms as they see fit given their respective priorities. P/T governments fund healthcare systems from general taxation, which is partly from their own province or territory and partly from the federal government. The federal government transfers their portion of funds to P/T governments, conditional on their compliance with the five principles of the 1984 Canada Health Act, namely public administration, comprehensiveness, universality, portability and accessibility [1]. The proportion of total health spending covered by the Canada Health Transfer (i.e. from the federal government) has fallen from an original 50% to about 23% in 2019 [2]. However, there was also a transfer of tax points during that period from the federal government to provinces, which had the effect of transferring some of the tax collection and hence increasing the provinces’ coffers, partially compensating for the reduction in transfers. There are also other federal transfers that are directed to specific programmes such as mental health. All medically necessary hospital and physician care is covered without fees at the point of service by a single public insurer in each P/T that is administered by the government. Coverage for non-physician/hospital services (e.g. physiotherapy) varies across provinces. Cost control policies such as those aimed at shortening the hospital length of stay may translate into shifting costs to different players as services (and drugs) not delivered in the hospital may cease to be covered by the public insurer [3].

In 2003 and 2004, additional federal funding was announced to address what were considered unacceptable wait times for some healthcare services [4, 5]. While hospitals were almost exclusively funded with global budgets, some provinces took this additional funding as an opportunity to introduce what Ontario and Quebec called patient-based funding (hereafter, PBF). A range of funding reforms may be considered “patient-based”, including activity-based funding (ABF) and pay-for-performance (P4P), which are among the reforms considered in our research. On one hand, global budgets were viewed by some bureaucrats as lacking transparency, and lacking incentives to address efficiency, productivity and quality [6, 7]. They have been associated with increased wait lists [8] and restricted access to some services [9, 10]. On the other hand, some decision-makers considered introducing PBF as a strategy to increase volume of services and reduce costs and wait times for said services, in addition to providing greater transparency, reducing length of stay and improving efficiency [11].

PBF programmes differ from global budgets by the close link that they create between the funding and the delivery of services [12]. The funding allocated to healthcare providers is directly related to the characteristics of the services delivered, aligning it with the patient’s consumption of services. Various PBF models exist, such as ABF and P4P.

ABF is a terminology used internationally that is based on the United States’ diagnosis-related group (DRG) funding system in which providers receive a payment for each service delivered, with prices for each type of service predetermined. Hence, funding amounts are the result of the volume and prices of services delivered. Although it can appear as a retrospective payment, it can be used prospectively with end-of-year reconciliation for volumes of services provided. It is important to note that there is no pure ABF model that is applicable in all hospital settings. The application is highly customized to account for specific situations and allow better results [13]. Most countries use a mix of ABF and other funding methods to reduce the frequency and the extent of unwanted effects mentioned above [14].

In P4P, hospitals receive funding conditional on their achievement of predetermined criteria of performance; targets can be set for indicators of quality of care, volume or efficiency [15].

One of the key elements identified as necessary for the implementation of ABF and P4P is having a standardized set of metrics and collection of data which may require complex risk adjustment approaches to ensure fair and equitable funding [12, 16].

Among provinces that introduced these alternative PBF models, Quebec and Ontario phased in different variations. Quebec’s PBF strategy aimed to create different independent programmes for specific procedures, funding them separately. Procedures not included in these programmes were still funded through global budgets. Ontario implemented its own unique version of PBF, called quality-based procedures (QBPs), across the hospital system [7, 17, 18], implementing PBF more cohesively at a broader system level. The Ontario Health System Funding Reform (HSFR) was gradually introduced after the passing, in 2010, of the Excellent Care for All Act, and it was meant to better reflect the needs of the population, allocate healthcare funding more equitably, achieve better quality of care and improve outcomes, and moderate spending growth to more sustainable levels [17]. Although there are multiple systematic reviews on the effects of ABF and P4P on various outcomes such as healthcare utilization (e.g., hospital readmissions, length of stay) or mortality [11, 15, 19–21], there is less literature on the implementation process of these funding mechanisms. One systematic review of the implementation processes related to P4P suggests the need for regular programme evaluation and making changes to ensure continuous alignment with organizational priorities [22]. In a systematic review of the experience of leaders implementing ABF or P4P, Baxter et al. identify prerequisites for successful implementation as commitment from the healthcare organizations and from leaders [23]. The review also identifies lack of resources and lack of leadership as barriers to success [23].

Ontario and Quebec are often compared because they have some similarities, and they cover together about two thirds of the Canadian population. Their healthcare spending per capita and proportion living in urban areas are comparable [24]. However, there are important structural differences in healthcare systems; hospitals in Quebec are quasi-public while those in Ontario are private not-for profit organizations. The organizational structures of the provinces’ health systems have changed since 2005, with a shift towards regionalization and centralization which could affect funding mechanisms that require collaboration between healthcare sectors. In 2006, Ontario created 14 Local Health Integration Networks (LHINs), and part of their mandate included the allocation of funds to hospitals in their respective geographical areas. Critiques of the reform argue that it did not enable integrated care [25]. Quebec had created regional health authorities (RHAs) in 1989, merged health and social services organizations together in 2006, and then implemented a major centralization reform in 2015, in which RHAs were abolished and health and social services organizations were further merged [26, 27]. In both provinces, most large academic tertiary- and quaternary-care hospitals remained independent entities. Generally, academic hospitals in Ontario do have more autonomy than those in Quebec, where chief executive officers (CEOs) and board members are appointed by the Ministry of Health and Social Services.

In reviewing the approaches, we aim to identify factors that supported or limited implementation, to inform future decisions regarding the use of PBF models in both provinces.

The objective of this study is to critically review PBF implementation approaches used by the two most populous provinces of Canada, Quebec (population: 8.5 million) and Ontario (population: 14.6 million), over 15 years.

## Methods

We used a narrative review approach to document and critically analyse Quebec and Ontario experiences with the implementation of PBF [28, 29]. We adopted this method, as narrative reviews are “scholarly summar(ies) along with interpretation and critique” [28, 29], to help us deepen our understanding of PBF through critical reflection of particular elements of PBF policy and implementation. Here, we define programmes as the “measures actually in place” [30].

### Data sources

We searched publicly available documents using Google and academic documents using the following electronic databases: PubMed, MEDLINE, EconLit, Web of Science and CINAHL. The databases were selected to cover a range of disciplines and collect information from economic, policy and public health perspectives. We used keywords specific to the funding programmes of the two provinces (see Appendix 1). The searches covered the period from 2003 (the year the Health Accord was signed) to 2019 (15 years after the introduction of the Health Accord). We selected this time frame for two reasons. First, this time frame allows to examine reform cycles from their inception to their implementation [31]. Second, it allows us to identify unintended and unexpected consequences of the policy reforms [31]. Publicly available documents included conference abstracts, theses, scientific papers, academic working papers, policy briefs, white papers, strategic papers and policy reports originating from presentations made in academic events, government reports and other relevant institutions. In addition, we contacted key stakeholders in health policy for additional documentation.

### Data analysis

**Fig. 1:**
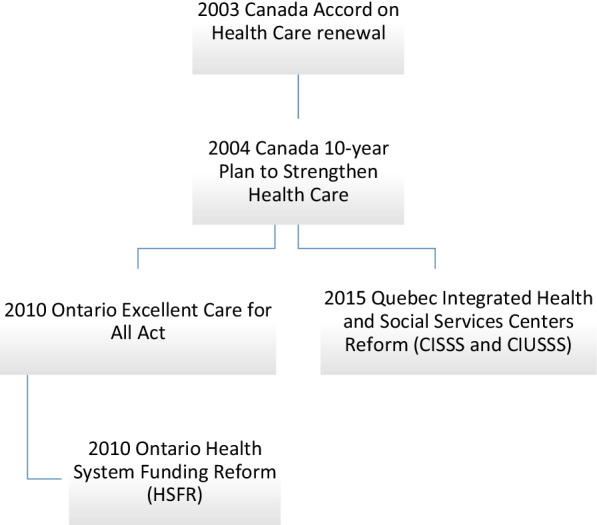
Pan-Canadian health policy reforms timeline

**Fig. 2:**
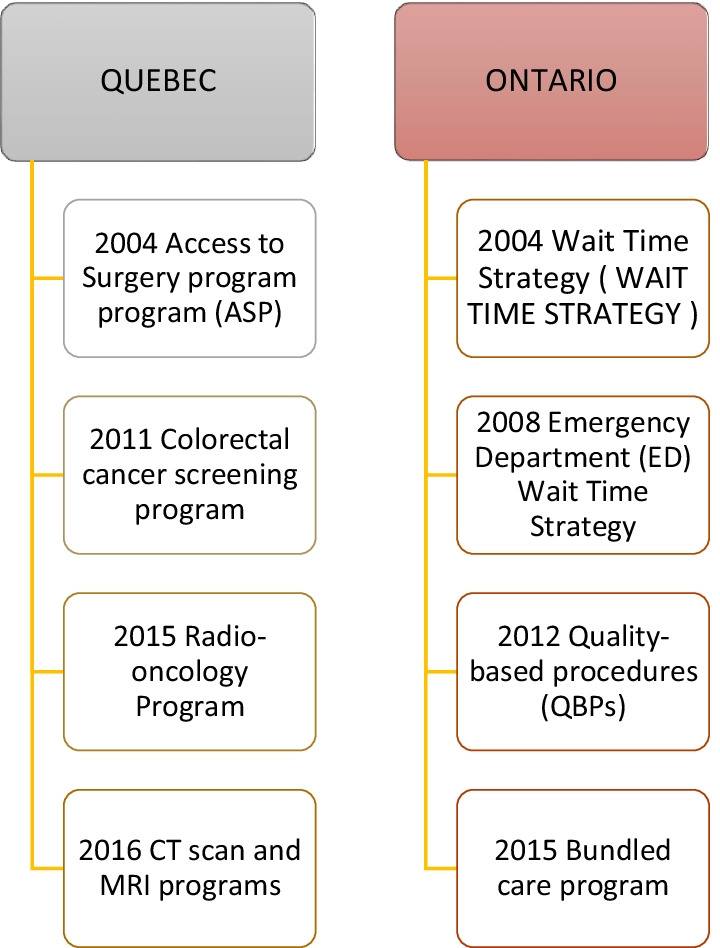
Quebec and Ontario PBF programme implementation timeline

To document the policy and implementation processes, we read, sorted and classified the documents per province, namely Quebec and Ontario, and identified their PBF programmes. We then created a timeline to identify key activities and documents according to health policy reforms (Fig. 1) and programme implementation (Fig. 2). We developed a data extraction sheet inductively informed by the initial scoping of the documents, including year, context, goals of the policy, dates of implementation, strategies implemented to achieve the goals, characteristics of the funding, unintended consequences and results. We used document analysis to extract and review the data [32, 33]. To do so, we purposively and judiciously selected and reviewed evidence from published and unpublished literature, paying attention to what was relevant to PBF implementation aspects [28].

## Results

We identified documents on PBF programmes in Quebec and Ontario, divided as follows: publicly available documents and presentations by government officials, scientific and academic articles focused on policy and implementation process or outcomes, press releases and reports. We report information on the different programmes in Table 1. Briefly, programmes aimed mostly at reducing wait times by incentivizing increased production of services (Quebec’s Access to Surgery programme, colorectal cancer [CRC] screening programme, and computed tomography [CT] and magnetic resonance imaging (MRI) programme; Ontario’s Wait Time Strategy [WTS] and Emergency Department [ED] WTS); and improving efficiency and quality of care (Quebec’s CRC screening programme and radio-oncology programme; Ontario’s QBPs and bundled care).

**Table 1:** Key elements of each programme in Quebec and Ontario

Quebec	Access to Surgery programme (ASP)	Colorectal cancer (CRC) screening programme	Radio-oncology programme	Computed tomography (CT) scans and magnetic resonance imaging (MRI) programmes
Year	2004	2011	2015	2016
Context	Response to the 2003 Accord on Health Care Renewal—Addressing wait times issues	2011, in response to the National Public Health Institute of Quebec (INSPQ) report on practice variations in colonoscopies		
ABF, P4P, or hybrid	ABF	P4P	ABF	ABF
Policy goals	Reduce wait lists and wait times	Improve performance, quality and access for CRC treatment and detect and treat the cancer before symptoms appeared	Improve efficiency	Improve access and reduce wait times
Implementation timeline	Modified in 2011	First introduced in eight pilot sites before being extended to all healthcare organizations in 2016	In 2016, the Ministry brought some modifications to the programme after hearing hospitals’ concerns. Modifications: Pricing was adjusted to reflect the first quartile of the average provincial costs (aiming at efficiency), adjusted by 2%. The 2% was a subjective measure. It also now includes salaries, benefits and social charges as well as maintenance and furniture costs Changes to the way volumes were calculated. The number of treatments to measure the volume of care was substituted by the number of hours of treatment	Modifications in 2018: Theoretical capacity was used for MRIs to set the minimal and optimal number of exams to be conducted in each work shift Actual operational hours and production objectives used to determine how many exams should be conducted during the day, evening and night shifts. Opening hours take into account weekends, holidays and maintenance
Implementation strategies	The additional funding associated with the ASP was given to regional health authorities, who then had to redistribute it to hospitals across their territories No funding limit given on the amount the hospitals could receive from the ASP No quality indicators were included	The CRC screening programme was implemented through the adoption of clinical guidelines and associated financial incentives The clinical guideline was developed in collaboration with the Ministry of Health and Social Services and their cancer branch. It stipulated that a faecal occult blood test (FOBT) should first be administered and that a colonoscopy should only be prescribed in the case of a positive FOBT Funding was allocated upon the achievement of volume and other performance targets There is no funding limit associated with this programme	Funding given to the hospital according to the volume of activity accomplished in a year	Each treatment price corresponds to the average provincial cost for the year 2014–2015, indexed each year. If a hospital has a negative volume compared to the baseline for one type of exam (CT or MRI), it will only receive funding if the global result is positive. If the additional total volume achieved for one exam (CT or MRI) does not compensate for the negative volume of the other (global result negative), no funding is allocated
Funding model characteristics	Additional funding was allocated to the providers upon achieving additional surgeries using the volume of 2002–2003 as the baseline	Performance criteria include: Targeted volume accomplished based on the number of colonoscopies achieved in 2010–2011 (and updated in 2014 to be the number achieved in 2014–2015) The production of at least 12 colonoscopies per room per day Funding was also conditional on the provider following the established guidelines and the quality standards, including as it relates to complications Additional funding is allocated for every unit of colonoscopy performed if all conditions are met. Pricing for each unit represents 100% of the average cost of a colonoscopy. The pricing of the treatment is based on the average total cost of human resources, supplies, sterilization, laboratory services and maintenance	It is a prospective payment with a holdback and a reconciliation process. An expected volume is calculated at the beginning of the year, and 90% of the funding is given in advance based on the unit price	An ABF model was put in place to allow healthcare providers access to more funding based on exceeding volumes of care compared to the ones achieved the previous year. The model was for direct operating costs only and did not include depreciation costs
Unintended consequences	Since only surgeries performed in the operating room (OR) were part of the programme, hospitals began using the OR for surgeries that did not require it. Tied to this issue, the categories were not specific enough to adequately reflect the costs of all the treatments that comprised them In 2011, the programme was modified to increase the number of categories from five to 16 and to include surgeries conducted outside of the OR as well. Although increasing the number of categories of surgeries improved the precision of the funding in relation to the operational costs, experts still considered the categories to be insufficiently precise. Additional measures were later implemented, including an information system, a definition of responsibilities regarding access to surgeries and a review process for the programme			
Results	Results of the ASP show both an increase in volume and a reduction in wait times in most categories. From 2002–2003 to 2012–2013, there was a 20% increase in volumes for all surgeries. With the introduction of new categories of surgeries, we can see changes for the period 2008–2009 for the different types of surgeries. There were no changes in mortality. Results show a wide variation in the percentage change in wait times in days, although this may be due to reporting in percentages rather than actual numbers The evolution of volumes was more volatile for hospitals outside of urban centres. An increase in volumes in those hospitals occurred until 2006, but they then dropped to lower levels than before the introduction of ASP. No information could be found to explain those results	The clinical standardization included in the programme contributed to decreasing the length of stay for patients in the hospital to an average of 2.2 days, as well as increasing the use of less invasive techniques. No effect was noted on the readmission or mortality rates. The financial incentive in itself was only found to decrease the hospitalization rate. Overall, from 2009–2010 to 2011–2012, the volume of colonoscopies increased by 4600 units each year, though this could be in part due to a temporary catch-up process of volumes. From 2010–2011 to 2012–2013, average wait times were reduced by 24 days	Results of the programme show an increase in efficiency. Spending increased due to a growth in volume, but efficiency gains reduced the cost per treatment. An increase in the hours of treatment declared after the modifications introduced in 2016 was also noted, which could be linked to an increase in quality since more time per patient allows for more precise diagnostics and more patient-centred treatments	No evaluation results were found on the effect of this programme

Ontario	Wait Time Strategy (WTS)	Emergency Department (ED) WTS	Quality-based procedures (QBPs)	Bundled care
Year	2004	2008	2012	2015
Context	2003 First Ministers' healthcare agreements	2006 Ministry commissioned a report to review the problem of overcrowding	After the 2010 Excellent Care for All Act, Ontario introduced PBF programmes under the system-wide Ontario Health System Funding Reform (HSFR)	Under the system-wide HSFR
ABF, P4P, or hybrid	ABF	P4P	Hybrid	ABF
Policy goals	Increase volume of services to reduce wait times in five key areas: cancer surgeries, cardiac revascularization procedures, cataract surgeries, hip and knee total joint replacement surgeries, and medical imaging [53, 55]	Reduce wait times, length of stay and crowding in the ED	Promote best practices	Strengthen home and community care
Implementation timeline	2004	The programme broadened through three waves: the first wave included 23 hospitals, the second 46 and the third 71	In 2012–2013, the QBP tariff corresponded to the 40th percentile of the 3-year average cost of treatment, excluding physician fees, in participating institutions. From 2013 onwards, it was changed to provincial average using the facility case mix index (CMI) as the price times the weighted cases for the volumes. A reconciliation process was also introduced	2015
Implementation strategies	CEOs and clinical leaders had to sign a purchase service agreement to receive funding. This agreement specified the responsibility of the hospitals in the maintenance of the baseline cases and the additional cases, the management of all wait times and the provision of wait times and quality information Accountability to the population increased with the use of a single wait time information system as well as a public forum on wait times Critical care improvement coaching available to help providers improve efficiency in service delivery Additional funding allocated for innovations and employee training Single rating scale introduced to help providers across Ontario determine the urgency of a patient’s condition Systemic savings made by purchasing CT and MRI equipment in bulk		Combination of pathways, analytical decisions and evidence to determine best practice and best cost P4P system that appeals to the hospitals’ aversion to loss, to encourage achievement of targets	It resembles the QBP funding programme, but it covers wider pathways that start when the decision for treatment is made and end after rehabilitation The pathway includes acute and post-acute care; partnerships needed to be created between providers
Funding model characteristics	ABF model to encourage a higher volume of care. It allocates additional funding to providers when they achieve more services than the baseline. Hospitals asked to volunteer the number of additional cases they could treat and to estimate their production cost. The final price per case set by a committee constituted of members from hospitals and the Ministry of Health and Long-Term Care (MOHLTC). It reflected full operational costs of the unit to ensure minimal impact of the increased volumes on other activities		Payment given up front to the providers but taken away if targets were not achieved. Performance targets changed depending on the wave, but they were always related to volume of care or patients’ length of stay in the ED. If the target was reached, providers were offered a fixed amount, except in the third wave, where a variable funding incentive was introduced [54, 56]. There was no competitive component between providers to access the funding Volume-based payments for QBPs represent 30% of funding	QBP prices were used to fund the part of the pathway that was the same as the QBP A price for the non-acute care included in the pathway was added to the QBP price. This non-acute care price is based on probabilities of different costs depending on the site where the care occurs
Unintended consequences	Even though overall results show a decrease in wait times for cancer surgeries, that was only the case for eight cancer groups. The other 11 groups showed an increased wait time average			
Results	Results of the programme show an increase in volumes. From August 2005 to April 2007, wait times also decreased in all areas, but targeted medically acceptable levels were only met in cancer and cardiac surgeries	Overall results of the programme indicate modest improvements. In the first year of implementation, three out of 23 hospitals participating in the programme met all targets. All waves considered, the program was associated with a reduction in overall wait times for admitted and non-admitted patients and in the percentage of patients leaving before being seen by a physician	Results of QBP implementation are varied depending on the QBP analysed. For example, the effects of the hip and knee arthroplasties were analysed at the London Health Sciences Centre, where patients were involved in the care process. Preadmission was intensified to better identify patients who needed medicine or anaesthesia consultations and plan their care accordingly. Strategies for patient education and early mobilization were also implemented to reduce hospital-acquired complications and readmissions. Results show a reduction in the length of stay of 1.7 days for hip and 1.8 days for knee arthroplasties, as well as an increase in overall patient satisfaction	Each programme was composed of a specific partnership between acute and post-acute care organizations, who were free to determine their clinical focus and set of services for the pathway. This made for considerable heterogeneity between the different programmes

The Ontario WTS uses an ABF model in order to encourage a higher volume of care [17]. It allocates additional funding to providers when they achieve more services than the baseline [16]. Hospitals were asked to volunteer the number of additional cases they could treat and to estimate their production cost [16]. The final price per case was then set by a committee comprising members from hospitals and the Ministry of Health and Long-Term Care (MOHLTC) [16]. It reflected full operational costs of the unit to ensure minimal impact of the increased volumes on other activities [16]. For the ED WTS, the funding incentive associated with this programme is a P4P system that appeals to the hospitals’ aversion to loss, to encourage achievement of targets [34]. The strategy was implemented in three waves. The payment is given up front to the providers, but is taken away if results are not sufficient [34]. The performance targets change depending on the wave, but they are always related to volume of care [17] or patient length of stay in the ED [34]. If the targets are reached, providers are offered a fixed payment, except in the third wave, where a variable funding incentive is introduced [34]. There was no competitive component between providers to access the funding [34].

The Ontario QBP programme consisted in establishing clinical pathways based on evidence of best practices, and a bundle cost for the episode of care corresponding to best practices. The prices were adjusted for patient complexity and included items corresponding to best practice such as rehabilitation after a hip surgery, but not readmissions [17]. The bundled care programme was introduced in 2015 [17] to help strengthen home and community care [35]. It resembles the QBP funding programme, but is set to cover wider pathways that start when the decision for treatment is made, and end after rehabilitation [17]. Since the pathway includes acute and post-acute care, partnerships need to be created between providers [36].

In Quebec, the Access to Surgery programme consisted in paying for each surgery performed above the hospital’s baseline of 2002–2003 [37]. There were five tariffs (hip, knee, cataract, other hospitalizations and other day surgeries) and no limit on the production volume. The CT and MRI programme was also based on a payment for each additional scan above the hospital’s baseline, except that the baseline for a given year was not static but instead corresponded to the volume performed by the hospital in the previous year [37]. Quebec’s CRC screening programme aimed at using the faecal occult blood test (FOBT) as the first diagnostic test so that only those with a positive FOBT would undergo a colonoscopy, rather than having the general population have colonoscopies, which was considered an inefficient use of resources [38, 39]. The funding for colonoscopies was conditional on meeting an annual average daily number of interventions per room, with a reduction in funding when the number was not met. For radio-oncology, the initial programme consisted of a payment for each patient started on a treatment, with a payment corresponding to the lower of a hospital’s cost or the provincial average cost. This was changed to a payment per hour of treatment with a tariff based on the first quartile [37].

All programmes had gone through some form of evaluation except for Quebec’s CT and MRI programme, which was more recent, and these evaluations led to modifications being made to limit unintended consequences. However, there does not seem to have been a systematic and formal evaluation of the implementation process and how healthcare organizations and providers reacted to the funding reforms in Quebec, in contrast to Ontario, where programmes were examined by independent researchers [18, 40, 41].

In our narrative review, we identified four factors that played a role in ensuring the successful—or not—implementation of these strategies: (1) adoption supports, (2) alignment with programme objectives, (3) funding incentives and (4) stakeholder engagement. Here, we outline how each factor supported or limited the implementation of these PBF programmes in Quebec and Ontario (see Table 2).

**Table 2:** Factors supporting or limiting PBF programme implementation in Quebec and Ontario

	Adoption supports	Alignment with policy and programme objectives	Funding and pricing strategy barriers	Key stakeholder engagement
Key features	Clinical guidelines Additional budget to support innovation and training Government direct purchase of equipment	Quality goals Volume goals Priority health areas	Unclear pricing systems Misalignment between surgery categories and prices Average costs defining pricing	Lack of key stakeholder, such as patients, physicians, and policy-makers, engagement
Programmes	CRC screening programme (QC) Quality-based procedures (ON) Wait time strategy (ON)	CRC screening programme (QC) Access to Surgery programme (QC) Wait time strategy (ON)	Access to Surgery programme (QC) Wait Time Strategy (ON) Quality-based procedures (ON)	Access to Surgery programme (QC) Quality-based procedures programme (ON) Wait Time Strategy (ON)
Quebec	CRC screening programme: Funding conditional on following best practice guidelines Additional budget for software innovation	CRC screening programme: Improvement of quality of care objectives ensured by funding conditional on quality measures (in this case, as defined by the clinical guidelines) Access to Surgery programme: Alignment with the 2003 Health Accords’ key health priority areas (namely cancer treatment, cardiac surgeries, joint replacement, cataract surgeries and diagnostic imaging)	Access to Surgery programme: Prices did not always reflect the actual cost of the surgeries Programme funding given to the regional authorities rather than to health organizations implementing the programme	Access to Surgery programme: Information system did not allow reconciliation and verification of data regarding the surgeries and the corresponding funding Physicians were disconnected from the cost and quality management
Ontario	Quality-based procedures programme and Bundled care programmes: Availability of clinical guidelines; however, funding not linked to them Wait Time Strategy programme: Additional budget to support innovation and staff training Government direct purchase: of CT and MRI equipment in bulk	Wait Time Strategy programme: Incentives for increasing volume of care Alignment with the 2003 Health Accords’ key health priority areas	Wait Time Strategy: The tariff set for each category of care based on prices volunteered by hospitals Quality-based procedures programme: pricing was the 40th percentile of the average costs incurred over a 3-year period, meaning that only the 60% less-performing institutions had the financial incentive to reduce their costs and increase their efficiency	Wait Time Strategy: Focus on empowering patients and accountability of healthcare providers Quality-based procedures programme: educational strategies to optimize the care and the cooperation between patients and caregivers In both programmes: Ministry of Health and Long-Term Care and different healthcare organizations as well as patients were consulted to fix the prices, to determine the care pathways or to plan the framework
Weaknesses	Limited integration of quality metrics into PBF models	Wait Time Strategy programme (ON): Lack of incentives for ensuring appropriateness of care In Quebec, the method used to calculate volume increase did not incentivize efficiency and sustainability across all programmes	Unclear funding and pricing strategies generated a perceived disconnect between the service provided and the financial reward	Not all programmes consistently engaged with relevant stakeholders Difficulties facilitating physician engagement [17] and encouraging communication between all actors

### Adoption supports

Quebec and Ontario presented a variety of adoption supports to help hospitals achieve PBF programme objectives. In both provinces, the governments supported PBF implementation in the form of targeted procedures or actions, such as the development and adoption of clinical guidelines, introduction of financial incentives and direct purchase of healthcare resources.

Clinical guidelines were used in Quebec’s CRC screening programme and in Ontario’s QBPs and bundled care programmes [17, 37, 42, 43]. In Quebec, the funding for CRC screening was conditional on following best practice guidelines [37, 38]. Clinical guidelines were considered a key contributor to the results observed by decreasing length of stay and promoting less invasive techniques [39]. Ontario, instead, developed its policies using expert panels and distributed clinical handbooks with evidence-based guidelines across the QBPs and bundled care programmes. However, funding was not linked to the implementation of clinical guidelines in practice [11]. Even though evidence regarding the effects of clinical guidelines adoption was not extensive, in some instances it seems to have helped reduce medical errors, increase efficiency and improve quality of care [38]. Overall, however, there was limited integration of quality metrics into PBF models.

Additional funding was allocated to both the CRC screening programme in Quebec and the WTS programme in Ontario [16, 38]. In Quebec, the added funding aimed at implementing clinical software to support and monitor activities [38]. In Ontario, it was used to help maintain innovation and for training staff [16]. The Ontario government also contributed to reducing the financial burden of hospitals by directly purchasing CT and MRI equipment in bulk [16], which supported implementation by allowing hospitals to conduct more exams.

### Alignment with policy and programme objectives

Quebec and Ontario implemented funding models aligned with each programme's objectives as well as with the overall Pan-Canadian policy reform goals. In the CRC screening programme (Quebec), quality incentives were given upon compliance with clinical guideline standards [37]. The use of clinical guidelines ensured that programme implementation aligned with quality objectives. In Ontario, incentives for increasing volume of care were introduced in the WTS programme [16]. These incentives compelled health professionals to increase the number of patients receiving treatment, thus reducing wait times [16].

The key priority areas defined by the Health Accord, namely cancer treatment, cardiac surgeries, joint replacement, cataract surgeries and diagnostic imaging [44], were addressed in the WTS programme (Ontario) and in the Access to Surgery programme (Quebec). However, in Quebec, the method used to calculate the volume increase did not incentivize efficiency and sustainability across all programmes. For instance, the baseline volumes for the Access to Surgery programme did not change over time [45].

### Funding incentives

As we have highlighted so far, financial incentives tied to quality and performance facilitated the implementation of PBF programmes and ensured that programme and policy goals were achieved. However, other funding and financial incentive strategies limited uptake as intended by the programmes. In the Access to Surgery programme (Quebec), prices did not always reflect the actual cost of surgeries due to the broad surgery classification system [12]. In addition, a number of surgeries were unnecessarily conducted in the operating room to receive additional funding [12, 37]. This was later addressed in the programme’s 2011 modifications that removed the requirement for the surgery to be conducted in an operating room [12, 37]. In Ontario, policy-makers were aware of the risk for upcoding inherent to ABF-based programmes [14], but upcoding was not observed in programme evaluations.

In both provinces, the pricing system did not always contribute to ensure efficient care. Efficiency had not been established explicitly as an objective in the early programme but became a concern for some bureaucrats when examining the effects of the early programmes and was considered in the design of the more recent programmes [37–39]. In Quebec, all four programmes used average provincial costs to determine the pricing of each service [37, 38, 46, 47]. Yet, average costs do not encourage efficiency or cost-saving actions. Rather, it encourages convergence to the mean and does not favour improvement in performance to reach optimal levels [14]. The radio-oncology programme (Quebec), however, used the lower of two average costs: the average real cost to the hospital and the provincial average costs. A hospital would then receive the lower of those two [37]. A hospital with an average cost above the provincial average cost would only receive the latter and would thus be encouraged to increase its efficiency, that is, to determine how to reduce its production costs so that they were in line with the provincial average. A hospital with an average cost under the provincial average cost would receive their average real costs. If the hospital did not maintain its efficiency (i.e. operating at a given average cost which was under the provincial average), it would still receive the amount corresponding to its average cost at baseline. As such, the hospital was incentivized to not increase its production cost. After modifications made to the radio-oncology programme in 2016 [37], the pricing was modified so that it would instead be based on the first quartile [37, 47]. The modification also took into consideration equipment maintenance costs and a case mix (albeit only as measured by the number of hours of treatment). In this programme, hospitals were encouraged to identify areas of inefficiency and suggest improvement strategies.

Ontario used various pricing strategies. For the WTS, the tariff set for each category of care by the expert advisory panel was based on the prices volunteered by hospitals [16]. It is unclear whether the final price was set below the average to encourage efficiency. For the QBPs, prices were initially set as the 40th percentile of the average costs incurred over a 3-year period [12]. This means that only the 60% lower-performing institutions had the financial incentive to reduce their costs and increase their efficiency. The prices were set subsequently to the provincial average with a facility case-mix adjustment as QBPs were introduced.

In both provinces, a lack of clarity regarding the funding incentive was also noted. In Quebec, the funding awarded for the Access to Surgery programme was given not to the hospitals but to the regional authorities. This caused a perceived disconnect between the service provided and the financial reward [45]. Even though information was given to regional agencies to help redistribute funding according to each hospital’s volume of care [46], this practice might have weakened the effect of the financial incentive. Such a distribution approach could encourage efficiency with a global budget. In Ontario, before QBPs programmes were implemented, some of the categories were covered by the Wait Time Surgery programme. The transition from an ABF model to the new QBPs programme was not well explained. Key actors either thought it was unintentional or were simply unaware that a transition had been made [18]. The ED WTS also had weaknesses. There were delays in the distribution of the incentive [48], contributing to creating uncertainty about the sustainability of the programme. Additionally, even though targets were not met by most of the hospitals, further incentives were added the next year [48]. Hospitals might not have had the time or the resources to meet those higher targets.

### Key stakeholder engagement

Leader and key stakeholder engagement is a major component in creating and implementing funding reforms [23]. Yet, in both Quebec and Ontario, key stakeholders were not engaged consistently throughout PBF programme implementation.

In Quebec, there is no overall PBF programme involving all actors (such as clinicians, healthcare managers and care coordinators). Each adopted different planning and implementation logics without being necessarily coherent with other efforts made to achieve the same goals elsewhere in the system [12]. In the Access to Surgery programme, information available to the providers regarding the implementation methodology was lacking (personal communication). The information system did not allow the reconciliation and verification of data regarding the surgeries and the corresponding funding [12]. Physicians were disconnected from cost and quality management [12]. However, policy-makers recognized the importance of engaging clinicians in the implementation of the radio-oncology programme (2015–2016) and considered that their engagement and the transparency in the communication with the stakeholders enabled a more successful implementation [37, 43]. A lack of involvement of actors was also noted in Ontario, particularly in the QBP programme, especially in terms of approaches to engage physicians throughout the hospital system [42, 49]. In the specific case of the orthopaedic QBP programme, there was also a lack of communication between providers and those responsible for the implementation of the QBPs programme at a province-wide level [23].

However, in Ontario, actors in the healthcare system and patients were more involved in some programmes. The WTS focused on increasing healthcare providers’ accountability [17]. The QBP programme had some difficulties facilitating physician engagement [17] and encouraging communication between all actors involved [23]. Nevertheless, patients were consulted to develop some QBPs. For example, the hip and knee arthroplasty QBP programme included educational strategies to optimize care and the cooperation between patients and caregivers. A strength of the WTS and of the QBP programme was involving multiple actors in the programme design, thus integrating various perspectives. Representatives from the MOHLTC and different healthcare organizations as well as patients were consulted to determine the prices, to define care pathways or to plan the framework.

## Discussion

Our study examined various PBF programmes implemented in Ontario and Quebec through a 15-year period during which healthcare systems have evolved and priorities may have changed. Key PBF implementation success factors identified in our study include stakeholder engagement and alignment of financial incentives, and the integration of clinical guidelines have also been identified as key to the wait times management in healthcare [50]. A Dutch study identified information asymmetry, worsening reputation of insurers, lack of trust, misaligned incentives in the hospital setting, hesitation to accept financial accountability and lack of start-up funding as barriers to the implementation of health funding reforms. Although the health systems are very different—for instance, the Netherlands has multiple insurers rather than a single public insurer—there are some similar findings. Notably, the Dutch study identified the lack of start-up funding as a barrier, which relates to the adoption supports in our findings [51]. Funding reforms may require that hospitals reorganize services for which they may need initial support, whether that support is financial or in terms of human resources or information systems. Having reliable information and decision support systems was identified as an important facilitator in other studies on the implementation of funding reforms [52, 53]. Information is also required for policy-makers to evaluate the programmes, monitor results and make adjustments [54]. Other studies suggest that funding reforms could be considered as an iterative process in which evaluation and communication between policy-makers and leaders of healthcare organizations lead to tweaking the original payment design [53, 55], which was also observed in our study. These findings are related to the engagement needed for a successful implementation. Leaders of healthcare organizations, including executives, physicians and managers, need to be champions of the reforms and see the potential benefits [23, 53].

One of the elements that was identified as an afterthought in the design of the programmes and that led to some modifications was the original omission of considering appropriateness of care. Although this was not identified as a key element in the success of the implementation, it is an important element for aligning incentives with policy objectives. Failure to account for appropriateness of care is a weakness that was observed elsewhere, for example, when global budgets were replaced with ABF to reduce wait lists. In the Netherlands, the introduction of ABF was associated with decreases in wait lists [51]. However, it also involved the abolition of funding caps, which is often inherent to ABF (i.e. healthcare organizations will be paid for every service without a limit to the quantity that can be provided—as was also the case in Ontario and Quebec). Abolishing caps enables systems to increase capacity and hence increase volumes of services provided. However, it was perceived as an inefficient strategy to solve wait lists and reach a supply and demand equilibrium, in part because of supplier-induced demand [56].

The interest in adopting PBF models in Ontario and Quebec was accompanied by multiple reports, detailing the advantages and pitfalls of such models as well as road maps and strategies for implementing them [12, 57]. The United States developed DRGs and started using them for their funding mechanism in the early 1980s [58]. There was an uptake of this approach by European countries in the 1980s and 1990s [11, 56, 59]. In Ontario and Quebec, incentives to increase the supply of services would enable an equilibrium to be reached between supply and demand. However, as was also seen in the Netherlands [56], this could have the unintended effect of increasing supplier-induced demand, translating into people receiving services (such as elective surgeries or diagnostic imaging) which may have previously been considered inappropriate [60].

In the Canadian context, where in-hospital care is provided free of charge to patients, while there is no standard for coverage of out-of-hospital care, hospitals could seek to shift their costs to the consumer or to private insurers. We can hypothesize that such behaviour may have happened. For instance, reducing length of stay means that patients return home earlier, thus reducing the costs to hospitals and to governments, particularly if governments do not increase funding for home care services. However, the incentive for this behaviour is not specific to PBF, as global budgets could similarly entice hospitals to reduce their costs through such shifting, unless the global budget is adjusted for the number of bed-days, in which case the hospitals are incentivized for longer lengths of stay. We did not, in our review, note such behaviour specifically associated with the implementation of PBF.

In summary, we identified four findings that enable the implementation of funding reforms, namely (1) adoption supports, (2) alignment with policy and programme objectives, (3) funding incentives and (4) key stakeholder engagement. Implementing a funding reform translates into changes for hospitals, which are complex organizations, and in which expected changes require support. In both provinces, how these factors were operationalized included both activities facilitating the implementation of PBF programmes, and missed opportunities, some of which were addressed in modifications of the funding models. Adoption supports can come in the form of guidelines that inform providers on the behavioural changes that are expected from the reform, for instance in terms of quality in clinical care. These supports will be most effective if well aligned with stated policy and programme objectives. Implementation is about the processes that need to be put in place to reach identified objectives, yet we observed that these objectives were not always well communicated. Funding reforms entails changes in the incentives to providers. All funding mechanisms bear inherent incentives that can influence the behaviour of providers. Some incentives may be very explicit while others are implicit. Establishing prices for services is complex, and the amounts and characteristics of the payment modalities (for instance the absence of caps) will send signals to the providers. The design of the incentives needs to be carefully considered to ensure that they are aligned with the desired behaviour and limit unintended consequences. Reforms can be well designed in theory but not well implemented in practice if the designers fail to integrate key stakeholders. Stakeholders should include those who will operationalize the implementation and whose behaviour may be affected by the reform. Depending on the context, key stakeholders can include hospital executives and managers, but also clinical leaders such as physician champions.

The approaches in Ontario and in Quebec were also different in stated objectives. Quebec closely tied its PBF implementation to new funding for additional activities to reduce wait times. There were no objectives to move some proportion of funding from global budgets into PBF. As such, PBF remained marginal as a proportion of hospitals’ revenues (under 5%—personal communication). In Ontario, the HSFR aimed to have 30% of hospitals funding from QBPs, but the proportion was only 15.2% in 2018 [17].

## Conclusion

Ontario and Quebec introduced PBF models to address wait lists in the context of additional funding which enabled an increase in capacity, and to improve health system efficiency. Yet the implementation of new funding models does not always yield the expected results. Our study suggests that this may be due to underlying factors that were not sufficiently considered, namely, adoption supports, an alignment with policy and programme objectives, funding and pricing strategy barriers, and key stakeholder engagement. As governments are formulating plans for expanding PBF or introducing new funding reforms, it is important that they consider these key elements.

## Appendix 1

### Search Keywords

**Table:**

Series #	Keywords
1	“patient-based funding” OR “patient-based costing” OR “activity-based funding” OR “activity-based costing” OR “performance-based funding” OR “pay for performance” OR PBF OR ABF OR P4P OR “hospital funding” OR “healthcare funding” OR “health care funding”
2	“wait time strategy” OR QBP OR “bundle care” OR “linking quality to funding” OR LQ2F OR “wait time” AND “emergency department” AND Ontario
3	« financement axé sur le patient» OR « financement à l’activité» OR « financement à la performance» OR FAA OR FAP OR « financement des hôpitaux» OR « financement des soins de santé» OR « financement du système de santé» AND Québec
4	« radio-oncologie» OR « accès à la chirurgie» OR PAC OR « tomodensitométrie» OR « imagerie médicale» OR TDM OR IRM OR « cancer colorectal» OR PQDCCR AND financement OR financement du système de santé AND Québec
5	“radio-oncology” OR “access to surgery” OR ASP OR tomography OR scanning OR CT OR CAT OR MRI OR “colorectal cancer screening” AND financing OR funding AND Quebec

## Abbreviations

ABF: Activity-based funding
CRC: Colorectal cancer
CT: Computed tomography
DRG: Diagnosis-related groups
HSFR: Health System Funding Reform
MRI: Magnetic resonance imaging
P/T: Province and territory
P4P: Pay-for-performance
PBF: Patient-based funding
QBP: Quality-based procedure
WTS: Wait Time Strategy

## Declarations

### Availability of data and materials

Documents are available (see references).
